# Analysis and description of the stages of *Aspergillus fumigatus* biofilm formation using scanning electron microscopy

**DOI:** 10.1186/s12866-016-0859-4

**Published:** 2016-10-18

**Authors:** Alejandra Itzel González-Ramírez, Adrián Ramírez-Granillo, María Gabriela Medina-Canales, Aída Verónica Rodríguez-Tovar, María Angeles Martínez-Rivera

**Affiliations:** 1 Laboratorio de Micología Médica, Departamento de Microbiología, Escuela Nacional de Ciencias Biológicas (ENCB), Instituto Politécnico Nacional (IPN), Carpio y Plan de Ayala s/n, Col. Casco de Santo Tomás, Del. Miguel Hidalgo, 11340 Mexico City, Mexico; 2 Unidad de Microscopía ENCB, Instituto Politécnico Nacional (IPN), 11340 Mexico City, Mexico; 3 Prolongación de Carpio y Plan de Ayala s/n, C.P. 11340 Ciudad de México, Mexico

**Keywords:** *Aspergillus fumigatus* biofilm, Stages of biofilm, Scanning electronic microscopy (SEM), Microhyphae

## Abstract

**Background:**

Biofilms are a highly structured consortia of microorganisms that adhere to a substrate and are encased within an extracellular matrix (ECM) that is produced by the organisms themselves. *Aspergillus fumigatus* is a biotechnological fungus that has a medical and phytopathogenic significance, and its biofilm occurs in both natural and artificial environments; therefore, studies on the stages observed in biofilm formation are of great significance due to the limited knowledge that exists on this specific topic and because there are multiple applications that are being carried out.

**Results:**

Growth curves were obtained from the soil and clinical isolates of the *A. fumigatus* biofilm formation. The optimal conditions for both of the isolates were inocula of 1 × 10^6^ conidia/mL, incubated at 28 °C during 24 h; these showed stages similar to those described in classic microbial growth: the lag, exponential, and stationary phases. However, the biofilms formed at 37 °C were uneven.

The *A. fumigatus* biofilm was similar regardless of the isolation source, but differences were presented according to the incubation temperature. The biofilm stages included the following: 1) adhesion to the plate surface (4 h), cell co-aggregation and exopolymeric substance (EPS) production; 2) conidial germination into hyphae (8-12 h), development, hyphal elongation, and expansion with channel formation (16-20 h); and 3) biofilm maturation as follows: mycelia development, hyphal layering networks, and channels formation, and high structural arrangement of the mycelia that included hyphal anastomosis and an extensive production of ECM (24 h); the ECM covered, surrounded and strengthened the mycelial arrangements, particular at 37 °C. In the clinical isolate, irregular fungal structures, such as microhyphae that are short and slender hyphae, occurred; 4) In cell dispersion, the soil isolate exhibited higher conidia than the clinical isolate, which had the capacity to germinate and generate new mycelia growth (24 h). In addition, we present images on the biofilm’s structural arrangement and chemical composition using fluorochromes to detect metabolic activity (FUNI) and mark molecules, such as chitin, DNA, mannose, glucose and proteins.

**Conclusions:**

To our knowledge, this is the first time that, *in vitro*, scanning electronic microscopy (SEM) images of the stages of *A. fumigatus* biofilm formation have been presented with a particular emphasis on the high hyphal organization and in diverse ECM to observe biofilm maturation.

## Background

A biofilm is a consortium of cell populations that adhere to a biotic or abiotic surface and embed in an extracellular matrix (ECM), and it is a complex mixture of biopolymers, such as polysaccharides, proteins, nucleic acids, and lipids. Biofilms comprise an adaptive response of microorganisms to internal and external conditions of the microhabitat surrounding them; thus, they express changes in cellular physiology with a differential expression of genes, and they present phenotypic, genetic and structural modifications [1–5].

Biofilms are greatly significant in natural systems and industrial processes. The study of the biofilm of *Aspergillus fumigatus* covers a wide spectrum from the point of view of agricultural, plant pathology, veterinary, and biotechnology, which can yield a variety of biochemical products, such as chemical additives in food, and cleaning products. This fungus is employed in biotechnological processes due to its metabolic versatility and its capacity to secrete enzymes, proteins, and other important industrial metabolites; biofilms can occur in both natural and artificial environments [4, 6–8]. In the medical field, biofilms form on medical devices, such as catheters, valves, and contact lenses, in which microorganisms are encased. In terms of nosocomial infections caused by different microorganisms, it has been estimated that 65 % of are biofilm origin [8–10]. Biofilms are more resistant to antibiotics than planktonic cells of the same species and are described as a protective anti-predator niche in nature and in host immune responses during infection. Therefore, biofilms are regarded as a virulence factor. Studies on the stages observed in biofilm formation in the filamentous fungi are of great interest due to the limited knowledge that exists on this specific topic and because there are multiple applications that are currently being carried out [10–16].

On the other hand, knowledge about biofilms has been extensively supported by the use of electron microscopy, which provides information on both biofilm structure and the diverse forms of ECM [6, 15–18]. Here, we present evidence, by scanning micrographics, depicting the stages of *A. fumigatus* biofilm formation, which include the following: 1) adhesion with cell co-aggregation and the secretion of exopolymeric substance (EPS); 2) the germination of conidia into hyphae and hyphae development with a high yield and expansion, and in the clinical isolate, irregular fungal structures, such as microhyphae, with short and slender hyphae, were observed; 3) biofilm maturation, hyphae and ECM, which form a complex structural arrangement; and finally, 4) cell dispersion.

## Results

### Microbiological and molecular identification

*Aspergillus fumigatus,* from the soil and from clinical isolates, was growth on potato dextrose agar (PDA) medium for five days at 37 °C. In both of these conditions, *Aspergillus fumigatus* developed the morphological features of this species as follows: velvety colonies with a flat surface; an anverse that was greenish-gray and reverse, colorless mycelia, and no presence of exudates and soluble pigments. The microscopic features included uniseriate *aspergilli*, columnar conidial heads, flask-shaped vesicles with the phialides covering one half to three quarters of the vesicle, and globose conidia that were finely rough and plain green in color [19, 20]. The molecular identification of *A. fumigatus* was performed, and basic local alignment search tool (BLASTN) analysis of the nucleotide sequence of the internal transcribed space (ITS) fragment of (600 bp) revealed 100 % homology with the sequences reported from *A. fumigatus* in GenBank for both of the isolates [21].

### Biofilms growth curves

**Fig. 1:**
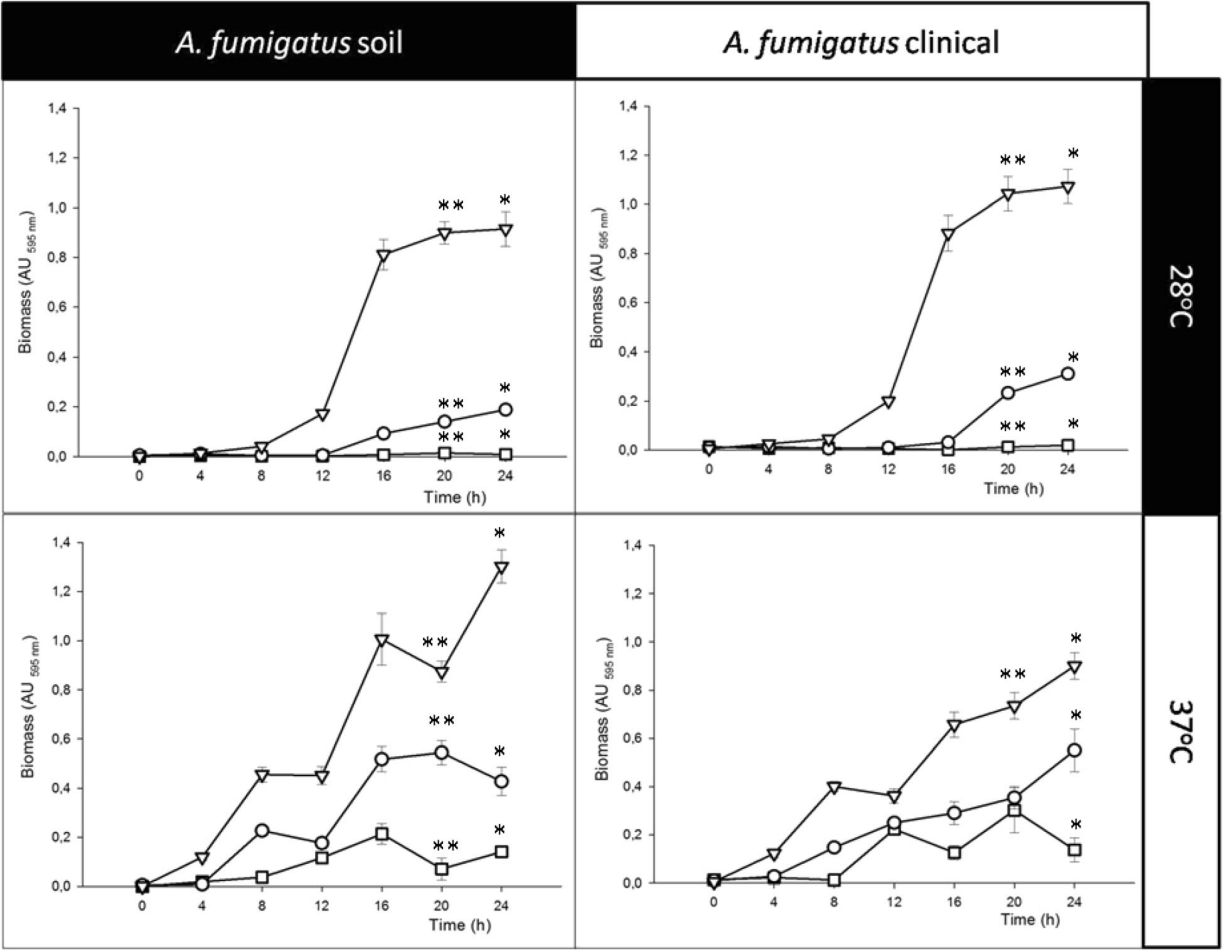
*Aspergillus fumigatus* isolates biofilm growth curves. Biofilm growth was assessed at different inoculum concentrations ranging from 1 × 10^4^ (square), 1 × 10^5^ (triangle) to 1 × 10^6^ (circle) and various incubation times (0, 4, 8, 12, 16, 20 and 24 h) and at two dissimilar temperatures (28 and 37 °C). In addition, two strains isolated from different niches were used (clinical and soil isolates). Significant differences were determined by a Student-Newman-Keuls test, performing a multicomparison of procedures (*p* < 0.050). The significant difference is described as follows: *comparison of the inocula of the biofilm at 24 h; **Comparison among the inocula at 20 h

Biofilm formation was carried out for *A. fumigatus* (soil and clinical isolates) utilizing different conditions: inoculum concentration, temperature and incubation time (Fig. 1). Both of the isolates of *A. fumigatus,* with inocula of 1 × 10^6^ conidia/mL at 28 °C during 24 h, demonstrated stages similar to those described in the classic microbial growth: the lag, exponential, and stationary phases (Fig. 1). *A. fumigatus* biofilm formation was similar between the two isolates. However, biofilm formation was slow and stable at 28 °C, while, at 37 °C, biofilm development was fast and uneven in both of the isolates. The optimal conditions for a high biofilm-stable production in both of the isolates were the following: 1 × 10^6^ conidia/mL at 28 °C during a 24 h incubation (Fig. 1).

### Stages of bofilms by SEM

**Fig. 2:**
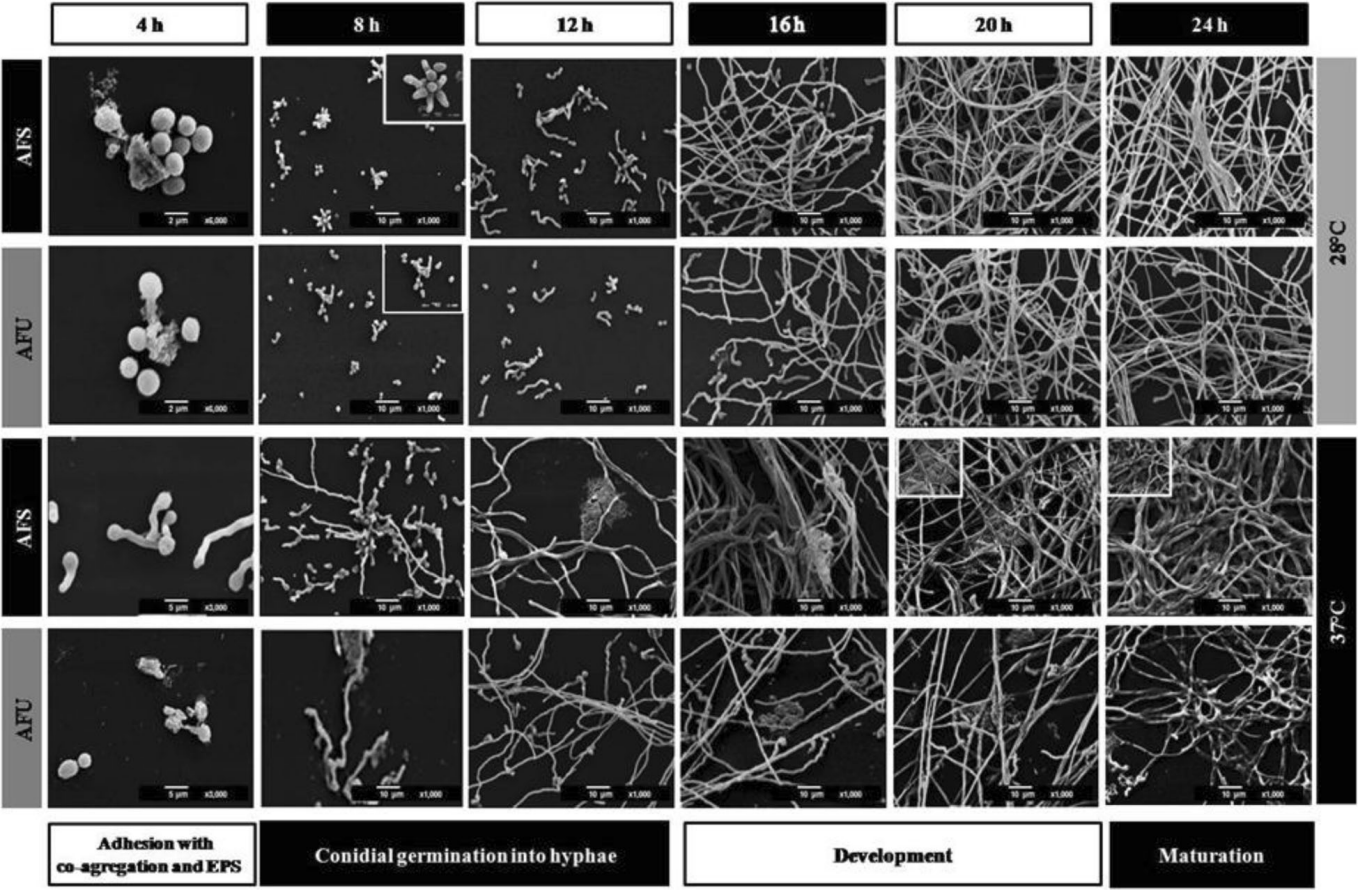
Stages of the *Aspergillus fumigatus* biofilm isolates. These were described by scanning electronic microscopy (SEM) in both of the *A. fumigatus* isolates: clinical (AFU) and soil isolates (AFS). Inoculum concentration 1 × 10^6^ conidia/mL, and in various stages of biofilm formation was perceived: **i)** Adhesion with co-aggregation and exopolymeric substance (EPS) production, 3,000X and 6,000X (4 h); **ii)** Conidial germination into hyphae, 1,000X (8-12 h) and development, 1000X (16-20 h); and **iii)** Biofilm maturation, 1,000X (24 h). Blank box, extreme left side: details increasing (3,000X-6,000X)
(ii) Conidial germination into hyphae and development (8-12 h incubation). The conidia was extended in several branching hyphae, and anastomosis was initiated at 37 °C; however, at 28 °C, the cell aggregation was more active and presented cellular organization (microcolonies) (Fig. 2 (8-12 h)). Biofilm development (16-20 h): the hyphae extended and formed networks (at both incubation temperatures); at 37 °C the biofilm was more organized than at 28 °C. The EMC was condensed and was expanded among the hyphae, and anastomosis was more evident only at 37 °C. Finally, for both of the temperatures, channel formation was observed during the initial phases (Fig. 2 (16-20 h)). In the late stage of biofilm development, irregular fungal structures, such as microhyphae, with short and slender hyphae were observed in the clinical isolate (Fig. 4).
(iii) Biofilm maturation (24 h). Mycelia development and expansion was more evident and included compacted hyphal layering networks, hypha-hypha adhesion, anastomosis at both temperatures (Fig. 3), and the formation of channels, but at 37 °C this channel was more evident. High structural arrangement and a wide production of ECM (only at 37 °C) were exhibited (Figs. 2/24 h, 3a-f ).

The stages observed during biofilm formation were the following:

(i) Adhesion, cell aggregation and EPS production (4 h incubation). During this stage, physical contact between the conidia and the surface and the conidium-conidium was executed. Moreover, early conidial co-aggregation occurred and EPS production was evident and was secreted at 28 °C and 37 °C (Fig. 2 (4 h incubation)).

**Fig. 3:**
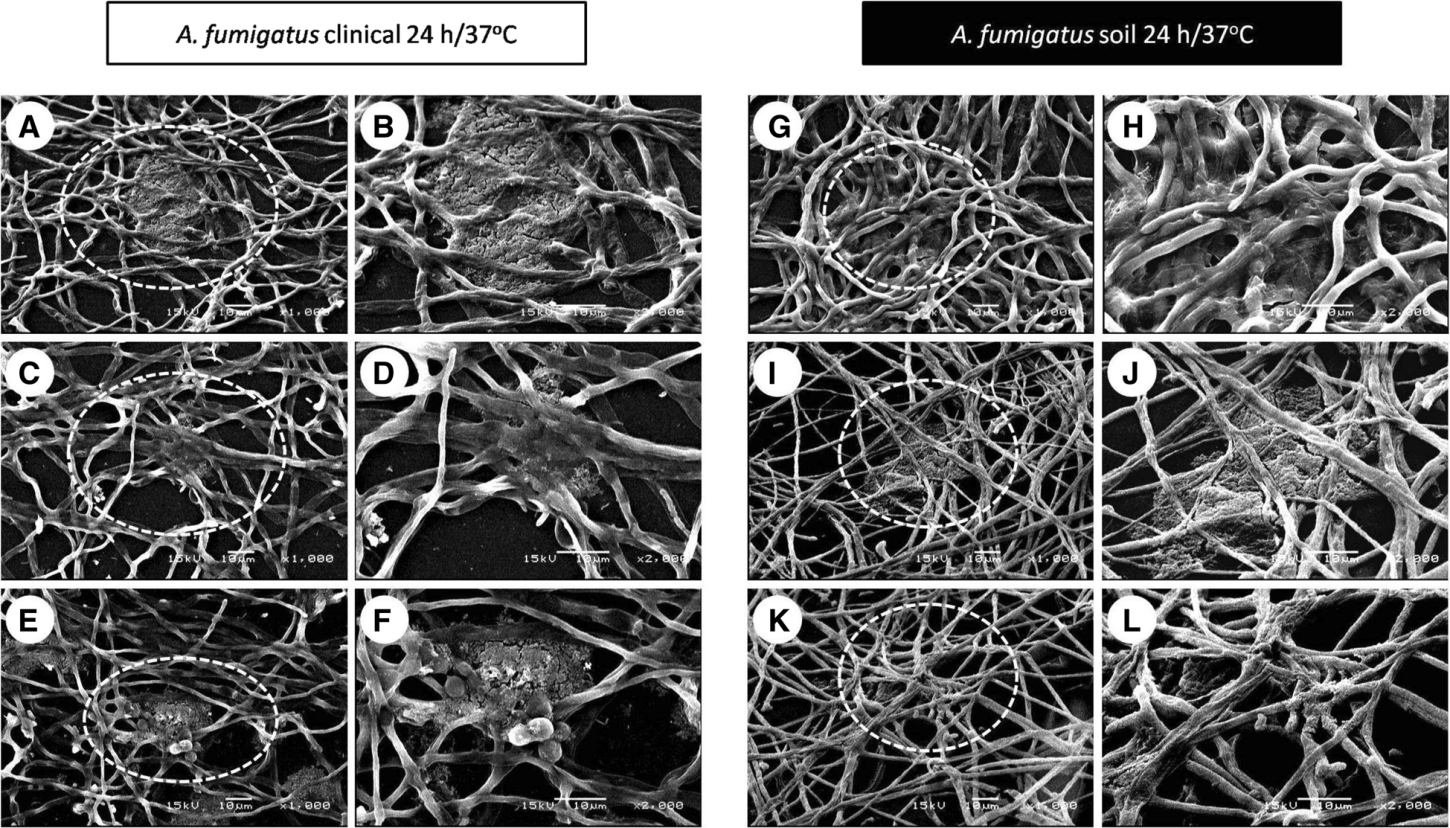
Extracellular matrix (ECM) structure of the *Aspergillus fumigatus* biofilm isolates. Inoculum concentration was 1x10^6^ and at 24 h/37 °C of incubation. The in vitro biofilm was observed by scanning electron microscopy (SEM) and certain types of ECM were differentiated. In the clinical biofilm isolate, a porous ECM (**a-b**: 1,000X-2,000X; **e-f**: 1,000X-2,000X) and condensed ECM (**c-d**: 1,000X-2,000X) was observed. Similarly, the same types of ECM for the soil biofilm isolate were observed. Porous ECM (**i-j**: 1,000X-2,000X) and condensed ECM (**g-h**: 1,000X-2,000X; **k-l**: 1,000X-2,000X). White dotted circles: ECM areas were observed at a higher magnification in the image on the right
(iv) Cell dispersion was observed only at the 24 h incubation time point at 37 °C. In certain, fields, biofilm formation was not homogeneous, and the presence of cell detachment, new conidia, and conidial germination was observed in both of the isolates; however, microhyphae were observed only in the clinical isolate (Fig. 6).

### Chemical composition of biofilm

The chemical composition of the biofilm was shown by epifluorescence microscopy (EPM). Biofilm *status* is 24 h/37 °C. The ECM components were analyzed by the co-localization of the fluorochromes as follows: a) chitin with Calcofluor white (green halo); b) metabolic activity with fluorescent vital staining dye probes (FUN1) (red halo and vacuoles); c) nucleic acids with fluorescent dye 6-DiAminidine 2-PhenylIndole (DAPI) (blue halo); and d) proteins with a Flamingo stain (magenta halo). The biofilm chemical composition is illustrated in Fig. 5a, with the bright field (without fluorochromes) presented to the right, and the Calcofluor white, FUN 1, DAPI and the overlapping of the fluorochromes are on the left. In Fig. 5b, the fields depicted are Calcofluor white and FUN 1, with the overlapping areas from left to right. Co-localization was evidenced by the presence of overlapping bands with more than two molecular components detected with bright yellow halos. Figures 5c and d depicts the spatial arrangement of the fungal biofilm constructed by confocal z-stack imaging analysis. Labeling with the fluorochromes for these images were Calcofluor white, FUN 1 and Flamingo stain. All of the fluorochromes, were co-localized in the ECM with hyphae embedded within it. The ECM showed a thickness of <10 μm (Fig. 5d)

## Discussion

From a medical perspective, *Aspergillus fumigatus* is an opportunistic pathogen of immunocompromised individuals, with a disease severity that depends on the host’s immune status, demonstrating a 50-95 % mortality rate. This fungus gives rise to local infections, such as nail dermatomycoses or fungal keratitis, and to invasive infections, such as aspergillosis, and comprises the second most common cause of fungal infections in hospitalized patients. *A. fumigatus* infection in the respiratory tract can cause lung fungal ball, invasive aspergillosis, invasive pulmonary aspergillosis (IPA), hypersensitivity pneumonitis, asthma, immunoglobulin E-mediated allergic rhinitis, chronic necrotizing pneumonia or allergic bronchopulmonary aspergillosis (ABPA). Additionally, it gives rise to osteomyelitis and endocarditis.

*A. fumigatus* develops a biofilm that may be one of the most important virulence factors [8–16]. *A. fumigatus* biofilm elaborates mycelia embedded in an EMC in vitro, and biofilm formation has been described in human bronchial epithelial (HBE) cells and cystic fibrosis bronchial epithelial cells (CFBECs) and in patients with cystic fibrosis [9, 10, 12]. Fungal biofilm formation on catheters and prostheses contributes to the development of nosocomial infections. Therefore, the persistence of fungal infections occurs due to the ability of a fungus to form biofilms on a wide variety of medical devices and because the persisting cells represent an important mechanism of resistance. The therapy subjected to an established biofilm in the host usually requires the administration of toxic concentrations of antimicrobials, and the recommended treatment includes the removal of the tainted device; however, this is a difficult and costly process. Therefore, fungal biofilms are a major clinical and economic problem [8–15, 22].

In the last decade, several studies have been published on the *A. fumigatus*’ biofilm, both in vivo (in murine models, in patients with invasive pulmonary aspergillosis and in primary human epithelial cultures) and in vitro (on polystyrene plates). In general, these studies mainly involve the biofilm maturity stage and the chemical composition of the ECM, with few images of the biofilm stages, but any one of these described all of the stages of biofilm formation. Thus, the information is different from the contributions made by our working group [5, 8, 11, 12, 17, 18, 23–28].

The most important contributions of this study are the following: i) we provide a description of every stages in the biofilm formation of *A. fumigatus* in vitro, over time, and the stages are supported with SEM images; ii) we analyzed two different origins of isolates: one from the environment and one from a patient with corneal ulcer; iii) we report micro-hyphae (clinical isolate) and fungal structures that have been scarcely reported to date and that have not been described, to our knowledge, for the *Aspergillus* species; and iv) we provide a description of the dispersion step for the formation of biofilm colonization at new points.

**Fig. 4:**
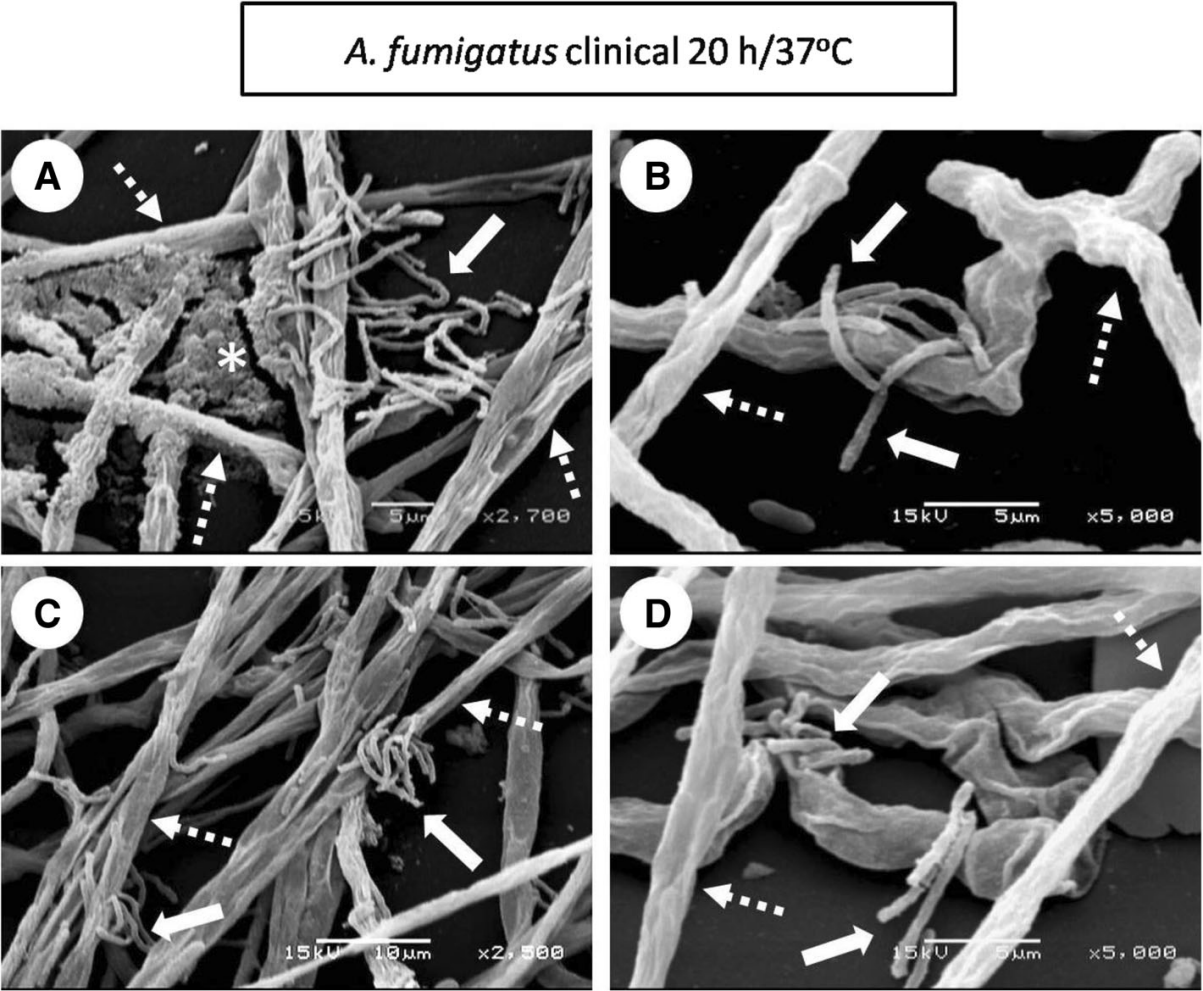
Microhyphae of the *Aspergillus fumigatus* clinical isolate biofilm. This structure was observed by scanning electron microscopy (SEM) at an inoculum concentration of 1 × 10^6^ conidia/mL only at 20 h/37 °C of incubation on the in vitro biofilm. Comparing the microhyphae with the size and diameter of the normal hyphae are crucial. **a** microhyphae projecting between the normal hyphae and the extracellular matrix (ECM) (2,700X); **b** microhyphae exiting and surrounding the normal hyphae (5,000X); **c** microhyphae on fungal anastomosis (2,500X). **d** developing microhyphae from the interior of the normal hyphae (5000X). White pointed arrow: normal hyphae; white arrow: microhyphae; white asterisk: porous ECM

To analyze the structural organization of the mature biofilm of *A. fumigatus* (24 h incubation at 28 °C and 37 °C), two strains, one from the soil and another from a patient with fungal keratitis, were examined by SEM. An overview of the *A. fumigatus* biofilm formation observed in this research was that these biofilms behaved similarly regardless of whether the isolate was from the soil or from the clinic; however, differences presented according to the incubation temperature. At 28 °C, the biofilm showed stages similar to those described in classic microbial growth: the lag, exponential, and stationary phases; biofilm growth was slow and stable with a low ECM production, and the fungal structural organization was simple (Fig. 1). At 37 °C, the performance curve showed a quite variable lag (adaptation) and log (exponential) phase, which could be in response to stress due to the incubation at a high temperature; thus, at 37 °C there is a reduction of the adaptation phase (lag) to maintain viable fungus; also, the log phase, with a discontinuous increase and with both behaviors is probably an adaptive response [29]. Thus, at 37 °C during the maturation stage, there was extremely organized mycelia structures, and these were reduced and compacted with hyphae that were thickened and fused into anastomosis, and the ECM was plentiful in its covering, surrounding, and strengthening fungal structures (Figs. 3 and 4).

In this study, we provide evidence of the *A. fumigatus* biofilm stages by SEM. The stages observed during biofilm formation were as follows:

### Adherence, cell co-aggregation and EPS production

At an early stage (Fig. 2/4 h), conidia adhere to the plate surface through an interaction of electrostatic forces among the structural components of the fungal cell wall, and this attraction force is weak and, therefore, reversible. Irreversible and permanent binding has been widely described in specific bacterial adhesins present on the cell surface, which bind to the substrate and EPS, which are substances produced by the microorganism in the initial stages of the biofilm formation that function in the adhesion of the cells to each other and to the substrate [8] and are composed of protein–carbohydrate complexes and glycoproteins that carry out mainly structural or adhesive functions. Adhesins are involved in the recognition of bacterial cells among themselves, including building bridges and initiating colony formation [3, 30, 31]. Adhesins are described in fungal adhesion during biofilm formation. In *Candida albicans*, *Candida glabrata* and *Candida tropicalis* biofilms, there is a group of adhesion genes involved in biofilm formation that are in the agglutinin-like sequence (*ALS*) family, which plays a key role in this process and encodes proteins possessing the characteristics of adhesin glycoproteins on the cell surface. The *ALS* family present in *C. albicans* includes eight genes (*ALS*1–*ALS*7 and *ALS*9) encoding many surface glycoproteins [32]. In *A. fumigatus*, six hydrophobin, comprising the rodlets RodAp, RodBp, RodCp, RodDp, RodEp and RodFp, have been identified on the surface of conidia. This hydrophobic characteristic permits the adhesion to proteins of host cells, and they could be involved in adhering to the surface of the polystyrene plate and initiating the process of biofilm formation in all or in only two or three of these [33]. Additionally, Gravelat and coworkers described this fungal interaction, and they found that adhesin MedA controls adherence to the polystyrene plate, biofilm formation, and the expression of conidiation genes and that it has hard effects on the conidiation process in *A. fumigatus* [33, 34]. Adhesion, resulting from the interaction between fungal adhesins and the plate surface, and adhesion conidium-conidium probably triggers signaling and promotes cell co-aggregation and EPS production, and these events are presented in Fig. 2 (4 h). At the same time, EPS accelerates fungal colony formation by the tight binding of the cells (Fig. 2 (8-12 h)) [34–37].

### Conidial germination into hyphae and development

Biofilm formation requires a threshold number of cells to enable them to be sensed and to generate a response, which is a regulatory mechanism of gene expression with specific functions [33]. In *A. fumigatus* biofilm formation, prior to beginning conidial germination, the conidia surface is markedly hydrophobic and is composed of 40 % hydrophobic methyl groups. *A. fumigatus* conidial germination results in the disruption of the hydrophobic proteinaceous-rodlet layer and reveals inner conidium walls that are essentially composed of polysaccharides, which are hydrophilic cell-wall components. There is a hydrophobic tip on a single germinating spore. The conidium loses its surface hydrophobicity progressively and, after that, the new growth-point exhibits a coexistence of hydrophobic rodlets and hydrophilic polysaccharides [33]. Conidial germination into hyphae begins with germ tube formation, as illustrated in Fig. 2 (8-12 h), possessing the very hydrophilic nature of the cell wall, and they are expected to favor hyphal growth [33].

### Biofilm maturation

*A. fumigatus* biofilm maturation was observed at 24 h, which is an incubation time similar to those reported by other researchers. The structural components include the ECM, which is present in the mature biofilm and binds the cells to form the structural base of the biofilm, including the EPS and many organized mycelia (Fig. 2 (24 h0) [15, 17, 18, 23, 24]. *ECM*. Water comprises the most abundant component and, in the biofilm, is nearly 97 %. In this moist environment, there is an ordered macromolecular network. The major functions described for EPS in bacterial biofilms are as follows: adhesion, cell aggregation, cohesion; water retention, a protective barrier as specific host defenses or antimicrobial agents, absorption of organic compounds and inorganic ions, enzymatic activity, nutrient source, exchange of genetic information, electron donor or acceptor, export of cell components, storage of excess energy retention, and the stabilization of enzymes [23, 24]. In fungal biofilms, all of these functions are not yet described, but some of these are being studied: the cohesive and adhesive forces of the matrix contribute to the architectural and mechanical stability of the biofilm. Fungal cells are immobilized into the matrix and act as a functioning ecosystem in continuously changing and homeostatically regulating with intense interactions, including cell-cell communication, which acts as the glue that holds the cells together [13, 15–17]. Biofilm structure greatly varies according to the microorganism producing it and the conditions surrounding its microhabitats, including the structural differences associated with the clinical presentation. During the infectious processes, the ECM supports protection against the host, as well as resistance to drugs by the microorganisms; thus, the ECM is not only a mechanical framework, but it is also a regulator of cell behavior. The matrix’s hydrophobic proteins are bound with the specific cell-surface receptors that result in the cell–matrix adhesion, which exerts an effect on cell shape, migration, proliferation, cell survival, and metabolism. In addition, the ECM protects the cells against environmental insults, including drying, Ultraviolet (UV), radiation, oxidation, starvation, the action of predators and host immune defenses and antibiotics [16–18]. The ECM characteristics were evident in Fig. 2 (24 h) and Fig. 3 and adhered to the fungal hyphae into a contiguous sheath and were also observed with a porous consistency (Fig. 2 (24 h)). In the *A. fumigatus* biofilm, the EPS was highly structurally arranged and had a plentiful production, which was covering, surrounding and strengthening fungal structures; it acts as a cohesive for fusing hyphae-hyphae structures (only 37 °C). The EPS occurs with a mucous appearance that adheres completely and covers the hyphae, causing anastomosis and closes the lumen of the water channels (Figs. 2 (24 h), 3, and 4). In previous studies, our working group described the *A. fumigatus* biofilm maturation stage, in which similar structures were observed [28].

**Fig. 5:**
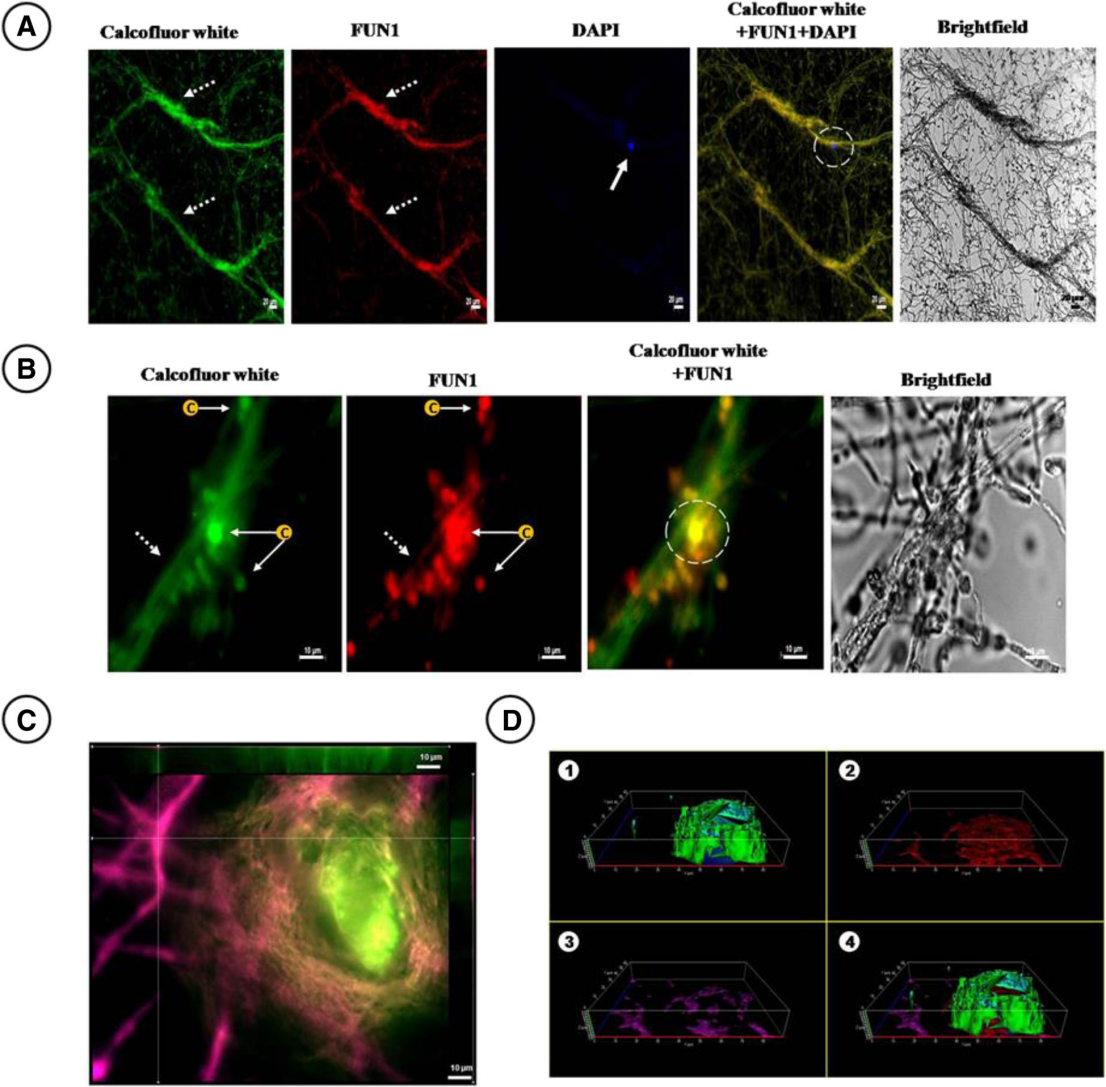
Structural composition of extracellular matrix (ECM) by epifluorescence microscopy (EPM). The EPM images on 12-wells polystyrene plates incubated with RPMI and an inoculum concentration of 1 × 10^6^ microconidia/mL at 24 h at 37 °C in vitro biofilm of *Aspergillus fumigatus* clinical isolate. **a** Co-localization of chitin/DNA. Hyphal anastomosis marked with Calcofluor white (chitin), FUN1 (fungal metabolic activity), and DAPI (DNA) (10X); **b** Detection of metabolic activity and chitin biofilm. Hyphal anastomosis indicated conidia marked with FUN1 and Calcofluor white. The latter exhibited a strong signal of chitin (100X); **c** Top view of the ECM detecting the co-localization of different molecules. Arrangement of z-stack images showing the dimensions of a section of the fungal biofilm, marked with FUN1, Calcofluor White and Flamingo stain; **d** Three-dimensional reconstruction of the in vitro biofilm showing molecular components of the ECM. These depicted different images that dissect the ECM and show some of its components marked as chitin (White Calcofluor, D1), hyphae with high metabolic activity (FUN1, D2) and protein (Flamingo, D3). In **d**, the merged image shows the reconstruction of the entire ECM model of the biofilm of *A. fumigatus*. All of the fluorochromes were co-localized in the ECM with the hyphae embedded within it. The ECM showed a thickness of <10 μm **d**. White pointed arrow: hyphae; white arrow: DNA (Signal with DAPI); white dotted circles: colocalization of exopolymers in ECM; c: conidia

In some micro-consortia, the chemical composition of EPS is known (carbohydrate polymers, DNA and/or proteins and, lipids, among others) but others remain to be identified. The *A. fumigatus*’-surface is composed of *α*-1,3-glucans, chitin, chitosan, galactomannan, galactosaminogalactan, melanin, and proteins. The composition and structural organization of the cell wall is constantly reshuffled; even though the polysaccharides present are the same, their amount and localization vary with the growth conditions and nutritional environment. Herein, we showed the chemical composition of *A. fumigatus* biofilm, which was observed by the co-localization of fluorochromes attached to chitin, metabolic activity and nucleic acids by CLSM; in addition, the overlap of the fluorochrome signals was observed when these attached two or three of these (Fig. 5). The function described for polysaccharides, such as α 1,3-glucans, comprised their playing a predominant role in vitro in the hyphal aggregation and in hyphal aggregation in biofilms. Other polysaccharides of the ECM, including galactomannan and galactosaminogalactan are also known to possess a role in protecting the fungus, and in the adhesion of its biofilm structures to surfaces [16, 25, 28, 35–37]. Extracellular DNA (eDNA) is an important component of the ECM biofilm that maintains the structural and architectural integrity of *A. fumigatus*. The eDNA is created by autolysis and has been significantly associated with the levels of antifungal resistance (Fig. 5). Furthermore, eDNA can be a reservoir of genes for horizontal gene transfer. DNA confers a more solid and resistant structural organization when it is co-localized with polysaccharides. eDNA derives from fungal cells due to the secretion of chitinases by *A. fumigatus* favoring its release (Fig. 5) [15, 23–25]. In biofilm, cell-wall modification exerts an essential impact on resistance to cell-wall drugs. In *A. fumigatus*, in a mouse biofilm model, in multidrug-resistant (MDR) efflux pumps *AfuMDR4* genes associated with the output of antimicrobials, the gene was significantly induced by treatment with Voriconazole after 24 h [8–12]. The FUN1 marker revealed metabolic activity that is a living community (Fig. 5).

*Mycelia*: The biofilm shows a complex 3-dimensional (3-D) structure that reflects a coordinated cellular process; mycelial development and expansion were evident, which included compacted hyphal-layering networks, hypha-hypha adhesion, anastomosis at both temperatures, with optimal spatial-arrangement-formed channels to provide influx of nutrients and efflux of waste products and thus stabilizes the biofilm; at 37 °C, this channel was more evident (Figs. 2 (24 h), 3, and 4). In addition, these structures were observed by other researchers [13, 16, 17, 23, 27]. *Microhyphae*: In the early stages of biofilm maturation, irregular fungal structures, such as microhyphae, in the clinical isolate, were observed (Fig. 4). This fact is relevant because there are scarce referrals to microhyphae in the literature, and this is the first time that they were described in *A. fumigatus.* Microhyphae present cytoskeleton alterations that generate short and slender hyphae with thin walls and with bent ends. Microhyphae are associated with a high enzymatic activity that favors the maturation process and the subsequent biofilm stage cell dispersion [38].

### Cell dispersion

**Fig. 6:**
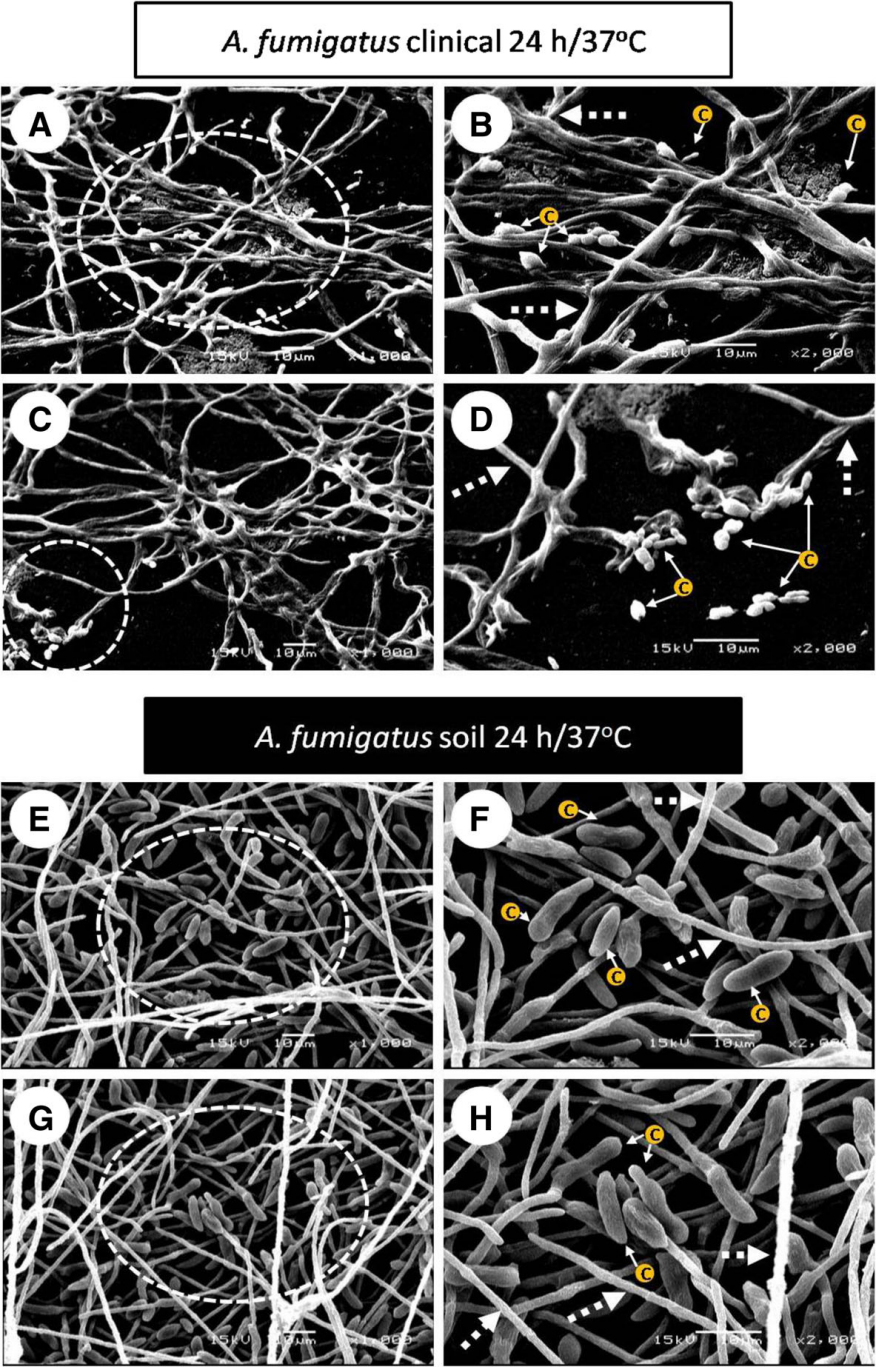
Cell dispersion stage of the *Aspergillus fumigatus* biofilm isolate. The latest phase of the biofilm formation of *A. fumigatus* was observed only at 37 °C for both of the isolates. Clinical isolate: **a-b** developing asynchronous conidia from the hyphae (1,000X-2,000X); **c-d** dispersion of the planktonic cells (1,000X-2,000X); Soil isolate: **e-f** presence of conidia released from mature biofilm (1,000X-2,000X). **g-h** conidial structure assembly during the cell-dispersion phase (1,000X-2,000X). White dotted circles: detailed presence of the dispersed fungal structures that are observed at a higher magnification in the image on the right; c: conidia

During cell-dispersion, a portion of the biofilm is detached, the portion comprising the conidia or hyphae. Asynchronous biofilm development was observed, especially at the biofilm-maturation stage when the new conidia were capable of germinating, producing new mycelial growth and hyphal modifications, such as curls (Figs. 4 and 6). Cell-dispersion of the biofilm occurs in response to environmental changes. This acts to remove a hazardous substance from the main body of the biofilm. This process leads to the dissemination and propagation of indwelling biofilm cells at a new location, which is supported by complex molecular events [17, 18]. Biofilms may be seen as protective shells of the living cells beneath, with extreme complex and countless functions, and thus, these are truly remarkable biological constructions. Biofilms provide protection against predation or chemical attack and provide inner-cells a medium for intracellular communication, nutrient flow, and the transfer of genetic material. The cell-dispersion disseminates viable cells to other locations in the environment or within a host where the cells can reproduce, thus, facilitating its persistence. Cell dispersion occurs as a result of scarce environmental nutrient conditions, and thus, it is a survival mechanism. Therefore, cell-dispersion is important not only for promoting genetic diversity but also for escaping unfavorable habitats, aiding in the development of new niches and the persistence of the microorganism at a new location [28, 36].

## Conclusion

The study of *Aspergillus fumigatus*’ biofilm covers a wide spectrum in terms of medical, agricultural, plant pathology, veterinary and biotechnological applications. Infections caused by biofilm-producing microorganisms are resistant to conventional antifungal therapy; thus, there is an urgent need to employ novel technologies and innovative therapies to achieve success in eradicating these microorganisms, and the knowledge of biofilm formation mechanisms is essential. The stages of biofilms have been described by in several studies, but, to our knowledge, they are not supported by micrographics of each stage as in the present study. Thus, the knowledge of the stages of *A. fumigatus* biofilm formation allows for its application in different fields.

## Methods

### Biological material

Two strains of *A. fumigatus* were used. One of these was isolated from the soil, and the other was a clinical isolate from patients with keratitis provided by the *Instituto de Oftalmología “Fundación Conde de Valenciana”* in Mexico City. Both of the strains were grown in potato dextrose agar (PDA) (BD Bioxon, México) medium at 37 °C for five days.

### Microbiological and molecular identification

*A. fumigatus* soil and the clinical isolates were identified by microbial methods: colony and microscopic morphology [14]. Colonial morphology was described on potato dextrose agar (PDA), and microscopic morphology was performed by the slide method as described by Johnson & Borman, [19, 20]. For molecular identification, genomic DNA was conducted using the Allers and Lichten method [39], and the ITS1-5.8S rDNA-ITS2 fragment was employed, following the amplification protocol described by Gardes, [21] for the two isolates as follow: The DNA was used as a template for amplification of the ITS1-5.8S rDNA-ITS2 fragment using the universal primers ITS1 (5′TCC CGT AGG TGA CCT GCG G 3′) and ITS4 (5′TCC TCC GCT TAT TGA TAT GC 3′) under the following thermal conditions: at 95 °C for 5 min; 35 cycles at 95 °C for 1 min; 50 °C 1 min; at 72 °C for 1 min; and at 72 °C for 10 min to final extension, following the protocol described by Gardes and Bruns [21]. The conditions for the PCR amplification were the following: 100 ng of genomic DNA as the template; 2.5 U of *Taq* DNA polymerase; 0.4 mM of Mg_2_Cl; 0.4 mM of dNTP in PCR Buffer Mix (Thermo Scientific®, Foster City, CA, USA) and 20 pmol of each primer. The reaction was performed in a MaxyGene™ II Thermal Cycler (Axygen®, Union City, CA, USA). The amplified fragments were visualized on agarose gels, with 0.5X TBE buffer (Trizma base, boric acid, EDTA [Sigma Chemical®, USA]) and were stained with ethidium bromide (Sigma Chemical®), using a StrataGene® transilluminator (StrataGene® Heidelberg, Germany). The PCR fragments were purified using a Zymo®DNA Clean and Concentrator TM-5’ Kit (Zymo®, Irvine, CA, USA) following the manufacturer’s instructions and were sequenced in the UbiPro platform (Facultad de Estudios Superiores Iztacala-UNAM) using the same primers. The nucleotide sequences were analyzed using the BLASTn tool (http://www.ncbi.nlm.nih.gov) to determine their identity.

### Biofilm growth

*A. fumigatus* biofilms were prepared on 96-well flat bottomed polystyrene plates (Nunc Roskilde, Denmark). They were performed using conidia harvested from the aerial static culture according to the method described by Mowat et al. [40]: *Aspergillus fumigatus’* conidia were suspended in RPMI as follow: the fungal aerial static culture was covered with PBS + 0.1 % Tween, and the surface was scraped with a glass rod bent for detaching the conidia. Next, the number of conidia recovered in 1 mL was quantified, and the inoculum for 1×10^6^ conidia/mL with sterile was adjusted with RPMI.

Biofilm formation was performed by the method described by Peeters et al., [41] and was quantified as described Christensen et al., [42] and was modified as by Ramírez-Granillo et al. [21, 36, 37]. It requires conidia adhesion for 4 h to remove the non-adherent planktonic cells, and 200 μL of fresh RPMI medium was added and biofilm formation was continued with (Ramírez-Granillo et al. [28]). Each assay was performed at least 12 times.

### Statistical analysis

The absorbance values of *A. fumigatus* biofilms were compared using a two-tailed Analysis of Variance (ANOVA); A Student-Newman-Keuls test was used to determine the significant differences employing SigmaPlot ver. 12.0 software (Systat Software Inc., San Jose, CA, USA).

### Analysis of biofilm structure by Scanning Electron Microscopy (SEM)

The *A. fumigatus* biofilms for electron microscopy were developed as described in the previous biofilm formation section, but 12-well polystyrene plates at 37 °C during 24 h (Santa Cruz Biotechnology, Santa Cruz, CA, USA) were used for this experiment. For the SEM, the samples were processed as described by Bozzola and Russell [43] and by Vázquez-Nin and Echeverría [44]. Briefly, the biofilms were washed with PBS and fixed with 2 % Glutaraldehyde (Electron Microscopy Sciences®, Washington PA, USA) for 2 h. Then, the biofilms were post-fixed with 1 % Osmium Tetroxide (Electron Microscopy Sciences®, Washington PA, USA) for 2 h. The bottoms of the 12- polystyrene plates were cut with a hot punch, and the intact biofilm was obtained. The samples were dehydrated with ethanol at 10, 20, 30, 40, 50, 60, 70, 80 and 90 % for 10 min and with absolute alcohol for 20 min. Then, the biofilms were placed into a critical point dryer and were coated with ionized gold for 400 s at 15,000 KV and 10 μA. The samples were observed in a scanning electron microscope (JEOL, Tokyo, Japan).

### Structural composition of the ECM by epifluorescent microscopy (EPM)

The biofilms were developed as previously described in 12-well polystyrene plates covered with a sterile coverslip (Velab, Mexico City, Mexico). The coverslips were recovered and placed in contact with a mixture of fluorochromes. The following fluorochromes were applied: Calcofluor white at 1 g/L (Sigma-Aldrich St. Louis, MO, USA) for chitin; FUN^®^1 at 10 mM (Life Technologies, Gaithersburg MD, USA) for metabolic activity; DAPI at 1.5 μg/mL (Vector Laboratories, CA, USA) for nucleic acids; and Flamingo® stain at 10X (Bio-Rad Laboratories Richmond, CA, USA) for proteins. The samples were observed under the epifluorescence microscope (Carl Zeiss, Germany) with the following filters: 480-530 nm (FUN®1); 360-460 nm (DAPI); 355-433 nm (Calcofluor white); and 512-535 nm (Flamingo®). The images and the three-dimensional reconstructions were processed with Zeiss LSM Image Brower ver. 4.0 software (Carl Zeiss, Germany).

## Abbreviations

ALS: Agglutinin-like sequence
BLASTN: Basic local alignment search tool
CLSM: Confocal laser scanning microscopy
DAPI: Fluorescent dye, 6-DiAmidino-2-PhenylIndole
ECM: ExtraCellular matrix
eDNA: Extracellular DNA
EPS: Extracellular polymeric substance
FUN-1: Fluorescent vital dye FUN®-1(2-chloro-4-[2,3-dihydro-3-methyl-{benzo-1,3-thiazol-2-yl}-methylidene]-1-phenylquinolinium iodide)
ITS: Internal transcribed space
PBS: Phosphate buffered saline
PDA: Potato dextrose agar
PI: Propidium iodide
SEM: Scanning electron microscopy
